# An Endoscopic Endonasal Approach for Early-Stage Olfactory Neuroblastoma: An Evaluation of 2 Cases with Minireview of Literature

**DOI:** 10.1155/2015/541026

**Published:** 2015-01-11

**Authors:** Hidenori Yokoi, Satoru Kodama, Yasunao Kogashiwa, Yuma Matsumoto, Yasuo Ohkura, Takayuki Nakagawa, Naoyuki Kohno

**Affiliations:** ^1^Department of Otolaryngology, Head and Neck Surgery, Kyorin University School of Medicine, 6-20-2 Shinkawa, Mitaka, Tokyo 181-8611, Japan; ^2^Department of Otolaryngology, Faculty of Medicine, Oita University, 1-1 Idaigaoka, Hazama-cho, Yufu, Oita 879-5593, Japan; ^3^Department of Pathology, Kyorin University School of Medicine, 6-20-2 Shinkawa, Mitaka, Tokyo 181-8611, Japan; ^4^Department of Otolaryngology, Head and Neck Surgery, Graduate School of Medicine, Kyoto University, 54 Shogoin Kawahara-cho, Sakyo-ku, Kyoto 606-8507, Japan

## Abstract

We describe the clinical findings in two patients with pathologically diagnosed olfactory neuroblastoma (ONB) of the sinonasal area and the surgical methods used for its treatment. Using an endoscopic endonasal approach (EEA) without dura resection, along with radiotherapy, we successfully treated ONB at the Kadish stage A. One of our patients, however, experienced tumor recurrence 24 years after open surgery with radiotherapy that was conducted at another hospital. This patient was no longer eligible for radiotherapy, and the tumor was therefore resected with dura resection using an EEA combined with duraplasty. The dura resection with duraplasty using fascia lata and a pedicled nasal septal flap was minimally invasive. As with surgery without duraplasty, a postoperative computed tomography (CT) examination revealed that EEA with duraplasty led to quick improvement of the postoperative inflammatory response as well as pneumocranium. Here, we investigated whether to modify the method of surgery depending upon the primary site of early-stage ONB. We suggest that, in early-stage ONB, an endoscopic endonasal approach is an effective and less invasive method. It is also advisable to perform dura mater resection of the lesion site despite the absence of obvious intracranial invasions in image findings.

## 1. Introduction

Recently, much progress has been made in the field of otolaryngology with regard to the use of endoscopic endonasal surgery. In recent years, this type of surgery has been successfully performed on patients with skull base tumors. It has also been reported that an extended endoscopic endonasal approach (EEA) is effective in the treatment of malignant sinonasal tumors such as olfactory neuroblastoma (ONB), chondrosarcoma, chordoma, early-stage squamous cell carcinoma, and adenocarcinoma [1]. Here, we investigated the benefits of modifying the method of surgery depending upon the primary site of early-stage ONB.

## 2. Cases

**Figure 1:**
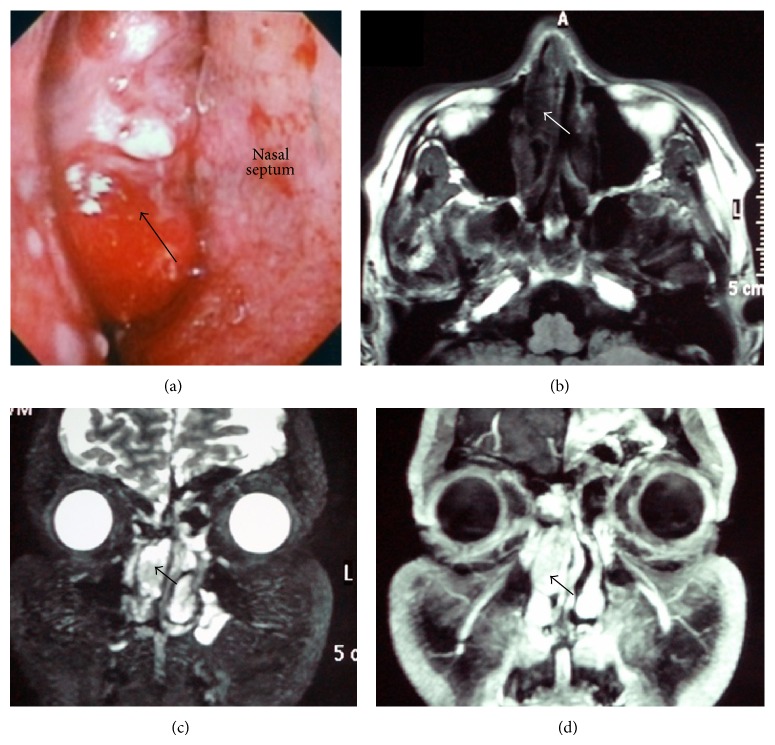
Preoperative local finding and MRI in case 1. (a) Tumor in the right nasal cavity (black arrow). (b) T1-weighted axial image showing low-intensity mass (white arrow). (c) T2-weighted coronal image showing a heterogeneous-intensity mass (black arrow). (d) T1-weighted coronal image (gadolinium+) showing contrast enhancement (black arrow).

*Case  1.* A 42-year-old man presented with a chief complaint of right-sided epistaxis and nasal stuffiness. He had no notable medical history. An anterior rhinoscopy revealed a dark-reddish tumour occupying the right nasal cavity (Figure 1(a)). A biopsy of the nasal cavity (performed under local anesthesia) led to a diagnosis of ONB.

A nonenhanced coronal computed tomography (CT) image showed a mass with a polyp-like appearance in the right nasal cavity, arising from the cribriform plate. No bone destruction was found. A contrast-enhanced coronal CT image detected a strongly enhanced mass. A magnetic resonance image (MRI) T1-weighted axial image revealed a low-intensity signal (Figure 1(b)), whereas a T2-weighted coronal image showed a clear heterogeneous mass and a T1-weighted coronal image provided a high-contrast image (Figures 1(c) and 1(d)). There was no evidence of the tumor having invaded the dura or intracranial space. No metastasis was evident from the examination of the positron emission tomography-CT (PET-CT). Therefore, the mass was diagnosed as an ONB in Kadish stage A.

**Figure 2:**
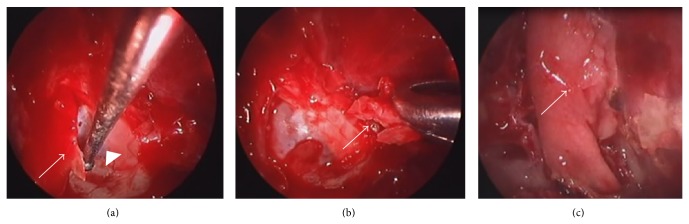
Intraoperative findings in case 1. (a) Only the cribriform plate (white arrow) was removed; biopsy of the dura mater (white arrowhead) was not performed. (b) It was confirmed through rapid intraoperative diagnosis that there was no obvious tumor infiltration in the cribriform plate mucosa, including a part of the olfactory glomeruli (white arrow), after tumor resection. (c) The dura mater was covered using a pedicled nasal septal flap (white arrow).

**Figure 3:**
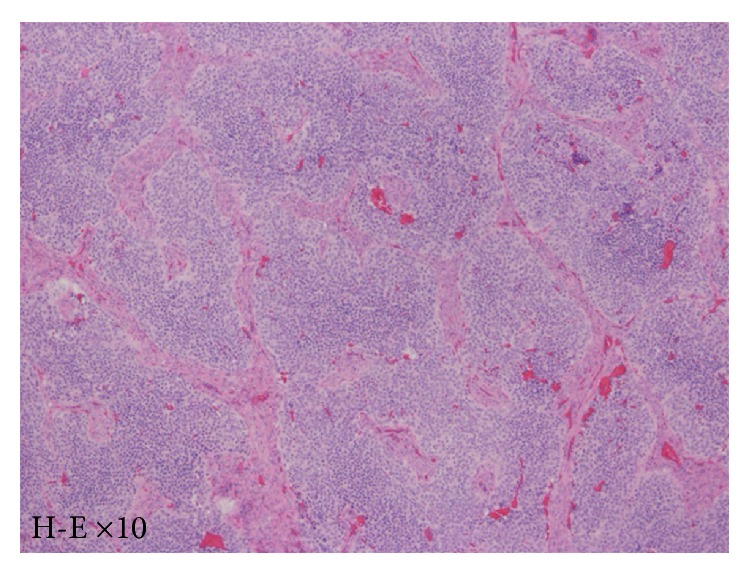
Pathological findings in case 1. Hematoxylin-eosin staining revealed a nest-like tumor mass under the mucosa. The tumor cells showed monotonous growth. Based on infrequent anisokaryosis and mitotic figures, the tumor was diagnosed as an ONB, Hyams's stage I-II.

**Figure 4:**
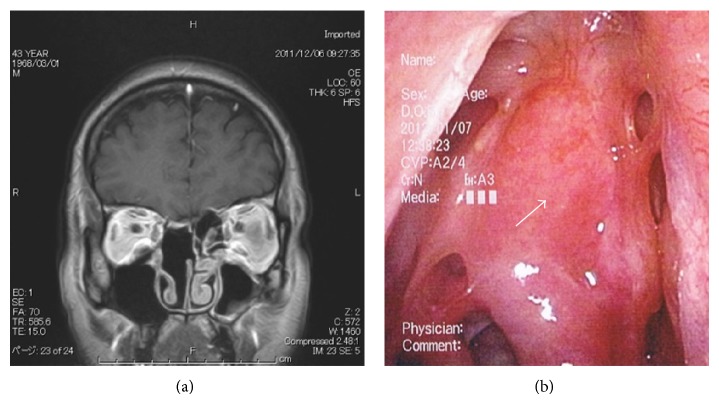
Postoperative MRI and local finding in case 1. (a) A T1-weighted coronal image (gadolinium+) showing no recurring mass. (b) The white arrow shows the pedicled nasal septal flap in the right nasal cavity.

Extirpation of the lesion by EEA was performed under general anesthesia. The tumor was resected from the base, the right ethmoid sinus was opened, and the middle nasal concha and superior nasal concha were removed. Intraoperative rapid diagnostic tests were performed as needed. The mucosa around the base of the tumor was then abraded from the base of the nose and the cribriform plate was removed. Following this, the dura mater was exposed (Figure 2(a)) and the fila olfactoria and surrounding mucosa were assessed using rapid diagnostic tests (Figure 2(b)). There was no evidence of residual tumor. Surgery was completed after a pedicled nasal septal flap was created to cover the dura mater (Figure 2(c)). Hematoxylin-eosin stains revealed a nest-like tumor mass under the mucosa. Tumor cells showed monotonous growth. Based on the infrequency of both anisokaryosis and mitotic figures, the tumor was diagnosed as an ONB, Hyams's grade I-II (Figure 3). Gamma knife irradiation at a dose of 18 Gy (a biological effective dose of 54 Gy) was performed 10 weeks after surgery. An MRI with contrast enhancement on the T1-weighted coronal image (Figure 4(a)), PET-CT scanning, and localized findings (Figure 4(b)) 50 months after surgery showed no evidence of tumor recurrence. 

**Figure 5:**
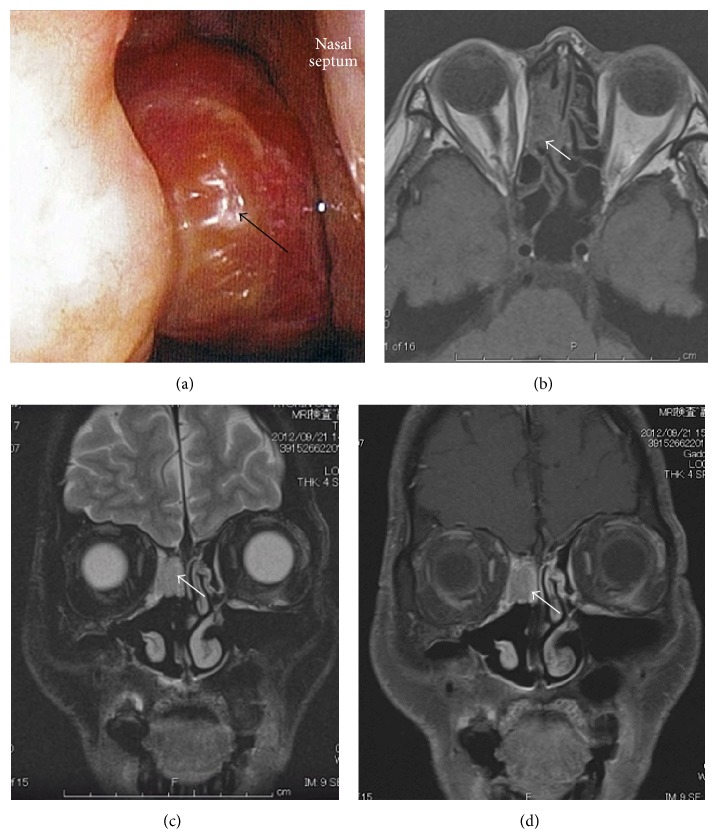
Preoperative local finding and MRI in case 2. (a) Tumor in the right nasal cavity showing a low-intensity area (black arrow). (b) T1-weighted axial image showing a slightly high-intensity area (white arrow). (c) T2-weighted coronal image (white arrow). (d) T1-weighted coronal image (gadolinium+) shows some enhancement within the lesion (white arrow).

*Case  2.* A 67-year-old man was diagnosed with an ONB (Kadish grade A-B) in 1989. He underwent lateral rhinotomy followed by irradiation and regularly completed follow-up visits at another hospital. At the end of August 2012, or 24 years after the initial tumor, the patient was referred to us for nosebleeds resulting from a mass in his right nasal cavity (Figure 5(a)). The tumor was biopsied under local anesthesia, and a diagnosis of recurrent ONB was made.

A coronal CT image showed the mass occupying the right anterior ethmoid sinus without a middle nasal turbinate. A contrast-enhanced axial CT scan showed patchy heterogeneous enhancement within the mass. A T1-weighted axial MRI showed a low-intensity area (Figure 5(b)), whereas a T2-weighted coronal image showed a slightly high-intensity area (Figure 5(c)). A contrast enhanced T1-weighted coronal image showed some enhancement within the lesion (Figure 5(d)). There was no evidence of the tumor having invaded the dura or intracranial space.

An operation by EEA was performed. The tumor relapsed 24 years after the first-line treatment (open surgery plus radiation) which made radiotherapy impossible; therefore, surgery was performed with a dural biopsy and duraplasty using fascia lata plus a pedicled nasal septal flap. First, biopsies were performed at 4 sites of about 15 mm surrounding the surgical stump; the safety margin was confirmed with intraoperative rapid diagnosis. The tumor with the mucosa around the base of the tumor was abraded from the mucosa at the base of the nose. The right anterior ethmoidal artery was clipped and cut to control blood flow from the mucosa at the cribriform plate to the dura mater.

**Figure 6:**
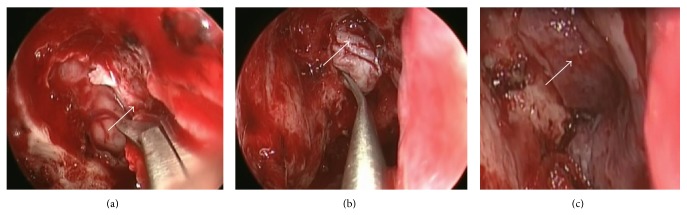
Intraoperative findings in case 2. Surgery was performed by dura resection ((a) white arrow) and duraplasty using fascia lata ((b) white arrow) plus a pedicled nasal septal flap ((c) white arrow).

**Figure 7:**
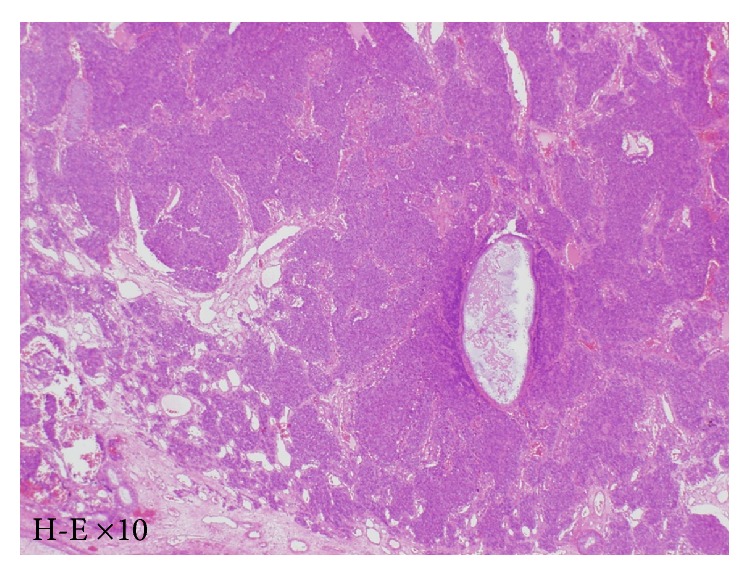
Pathological findings in case 2. Hematoxylin-eosin stains showed a nest-like tumor mass under the mucosa. The tumor cells showed monotonous growth. Based on slight anisokaryosis and mitotic figures, the tumor was diagnosed as an ONB, Hyams's stage II.

**Figure 8:**
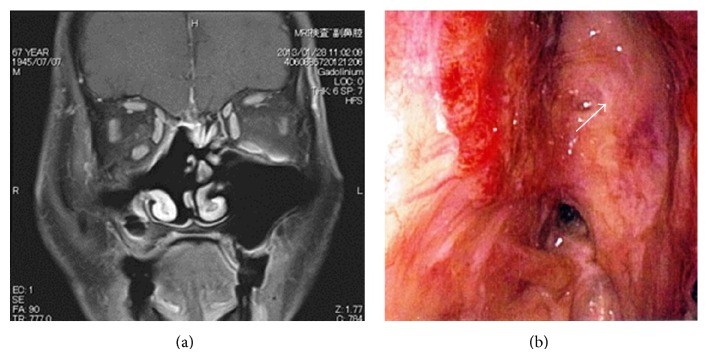
Postoperative MRI and local finding in case 2. (a) T1-weighted coronal image (gadolinium+) showing no recurring mass. (b) The white arrow shows the pedicled nasal septal flap in the right nasal cavity.

The mucosa at the cribriform plate was ablated, and the dura at the tumor base was resected (Figure 6(a)). After confirming that the dura mater was intact by using an intraoperative rapid diagnostic test, one piece of fascia lata was spread inside the dura mater in an underlay (Figure 6(b)), while another piece was implanted between the dura mater and the bones at the base of the skull. Surgery was completed after a pedicled nasal septal flap was created to cover the dura mater (Figure 6(c)). Hematoxylin-eosin stains showed a nest-like tumor mass under the mucosa. The tumor cells showed monotonous growth. Based on slight anisokaryosis and the presence of mitotic figures, the tumor was diagnosed as an ONB, Hyams's stage II (Figure 7). MR imaging (Figure 8(a)) and local findings (Figure 8(b)) 24 months after surgery showed no evidence of tumor recurrence.

## 3. Discussion

ONBs are thought to arise from the specialized sensory neuroepithelial olfactory cells that are normally found in the upper part of the nasal cavity, including the superior nasal concha, the upper part of septum, the roof of nose, and the cribriform plate of ethmoid [2]. Even though the tumor arises from the olfactory neuroepithelium, anosmia is not a common complaint (5% of cases). Due to the nonspecific nature of the initial presentation and slow growth of tumors, patients often have a long history before diagnosis [2]. The exact cell of origin of ONB is controversial [3]; proposed sources include Jacobson's vomeronasal organ, the sphenopalatine ganglion, the ectodermal olfactory placode, Loci's ganglion, autonomic ganglia in the nasal mucosa, and the olfactory epithelium [4].

ONB shows varying biological activity, ranging from indolent growth to a highly aggressive neoplasm [5]. This tumor constitutes 3% of all intranasal neoplasms [6] and occurs over a wide age range (from 3 to 90 years) with a bimodal peak in the second and sixth decades of life [7]. Both the Kadish surgical stage [7, 8] and Hyams's pathological stage [9] are predictive of survival.

The current recommended treatment strategy is surgery and, in selected Kadish stage A cases, this is combined with radiotherapy. Radiotherapy is conducted before or after surgery, at the primary site and cervical lymph nodes. Adjunctive chemotherapy may be added to this treatment depending on the degree of differentiation of the tumor for Kadish stage B. As for Kadish stages C and D, the suggested treatment strategy is preoperative chemotherapy and/or radiotherapy followed by surgery [10].

Recent findings have shown that sequential resection with negative margins is equivalent to open en bloc resection (Table 1) [11–16]. A comparison of survival results between 2002 and 2008 showed that the endoscopic surgery group maintained better survival rates than the open surgery group did (*P* = 0.0018) [17]. Although endoscopic methods have been associated with better survival outcomes, the overall follow-up was shorter after endoscopic surgery than after open surgery [17]. Furthermore, most tumors resected via open surgery were at Kadish stages C and D, whereas endoscopy and endoscopy-assisted techniques were more commonly used to remove Kadish stage A and B tumors. Endoscopic surgery is therefore still reserved for patients with less invasive lesions. Further studies are needed to differentiate between the outcomes of these surgical options [17].

It has been reported that olfactory neuroblastoma most commonly recurs within the first 4 years but can recur very late, for example, after 19.4 years in one case. There is currently no universally accepted follow-up regime, but even late recurrence of the disease is eminently treatable. Therefore, a protocol for lifelong follow-up with both clinical examination and serial imaging including the neck and entire intracranial compartment has been proposed [18].

In the 2 cases included in the present report, we performed first- and second-line EEA treatment for early-stage ONB. In the first case, dura biopsy plus duraplasty was not performed because residual mucosa in the cribriform plate area was intact in the intraoperative quick diagnostic test, and the current benign course was achieved, with gamma knife treatment for 50 months. In contrast, the second case showed tumor recurrence 24 years after craniofacial surgery plus radiotherapy. This patient was no longer eligible for radiotherapy, and the tumor was therefore resected including dura biopsy using an EEA combined with duraplasty to avoid microinvasion of the skull. To prevent a postoperative leak, we chose a dual repair procedure [19] and used a pedicle nasal septal flap [20]. The dura biopsy with duraplasty using fascia lata as well as a pedicled nasal septal flap was minimally invasive. As was the case with surgery without duraplasty, a postoperative CT examination revealed that these methods led to quick improvement of the postoperative inflammatory response as well as pneumocranium. This patient has had no recurrence for 24 months.

In this minireview, we examined whether the dura mater adjacent to the base of a tumor should be resected in EEA surgery for early-stage ONB. During surgery, it is most important to first determine a clear resection range and then to confirm this range in an intraoperative margin study. We believe that, during operations to treat recurrent ONB, the dura mater adjacent to the base of minimally invasive tumors should be resected.

## 4. Conclusion

We believe that, in early-stage ONB, EEA is an effective option. Additionally, it is advisable to perform dura mater biopsy of the lesion site despite the absence of obvious intracranial invasion in imaging findings. We propose a protocol for lifelong follow-up with both clinical examination and serial imaging.

## Figures and Tables

**Table 1:** A sequential resection with negative margins via endoscopic endonasal approach versus open en bloc resection.

	Number of cases (stages)	Resection	Period of observation (m)	Recurrence	Prognosis (%)
Unger et al. (2005) [11]	14 (B5, C9)	Piecemeal 2/en bloc 12	13–128 (58.0)	3/14	100
Castelnuovo et al. (2007) [12]	10 (A3, B4, C3)	Piecemeal	15–79 (37.0)	0/10	100
Suriano et al. (2007) [13]	9 (A3, B6)	Piecemeal/en bloc	26–60 (42.8)	0/9	100
Dave et al. (2007) [14]	9 (A5, B2, C2)	Piecemeal 3/en bloc 6	4–105 (36.7)	0/9	100
Zafereo et al. (2008) [15]	3 (A2, B1)	Piecemeal/en bloc	21–147 (67.3)	1/3	100
Folbe et al. (2009) [16]	17 (A2, B11, C4)	Piecemeal/en bloc	11–152 (45.2)	0/17	100
